# Neural Encoding of Active Multi-Sensing Enhances Perceptual Decision-Making via a Synergistic Cross-Modal Interaction

**DOI:** 10.1523/JNEUROSCI.0861-21.2022

**Published:** 2022-03-16

**Authors:** Ioannis Delis, Robin A.A. Ince, Paul Sajda, Qi Wang

**Affiliations:** ^1^School of Biomedical Sciences, University of Leeds, Leeds, LS2 9JT, United Kingdom; ^2^School of Psychology and Neuroscience, University of Glasgow, G12 8QQ, United Kingdom; ^3^Department of Biomedical Engineering, Columbia University, New York, New York 10027; ^4^Data Science Institute, Columbia University, New York, New York 10027

**Keywords:** active sensing, drift diffusion model, EEG, multisensory processing, partial information decomposition, perceptual decision-making

## Abstract

Most perceptual decisions rely on the active acquisition of evidence from the environment involving stimulation from multiple senses. However, our understanding of the neural mechanisms underlying this process is limited. Crucially, it remains elusive how different sensory representations interact in the formation of perceptual decisions. To answer these questions, we used an active sensing paradigm coupled with neuroimaging, multivariate analysis, and computational modeling to probe how the human brain processes multisensory information to make perceptual judgments. Participants of both sexes actively sensed to discriminate two texture stimuli using visual (V) or haptic (H) information or the two sensory cues together (VH). Crucially, information acquisition was under the participants' control, who could choose where to sample information from and for how long on each trial. To understand the neural underpinnings of this process, we first characterized where and when active sensory experience (movement patterns) is encoded in human brain activity (EEG) in the three sensory conditions. Then, to offer a neurocomputational account of active multisensory decision formation, we used these neural representations of active sensing to inform a drift diffusion model of decision-making behavior. This revealed a multisensory enhancement of the neural representation of active sensing, which led to faster and more accurate multisensory decisions. We then dissected the interactions between the V, H, and VH representations using a novel information-theoretic methodology. Ultimately, we identified a synergistic neural interaction between the two unisensory (V, H) representations over contralateral somatosensory and motor locations that predicted multisensory (VH) decision-making performance.

**SIGNIFICANCE STATEMENT** In real-world settings, perceptual decisions are made during active behaviors, such as crossing the road on a rainy night, and include information from different senses (e.g., car lights, slippery ground). Critically, it remains largely unknown how sensory evidence is combined and translated into perceptual decisions in such active scenarios. Here we address this knowledge gap. First, we show that the simultaneous exploration of information across senses (multi-sensing) enhances the neural encoding of active sensing movements. Second, the neural representation of active sensing modulates the evidence available for decision; and importantly, multi-sensing yields faster evidence accumulation. Finally, we identify a cross-modal interaction in the human brain that correlates with multisensory performance, constituting a putative neural mechanism for forging active multisensory perception.

## Introduction

In our daily lives, we make judgments based on noisy or incomplete information that we gather from our environment (Heekeren et al., 2004; Juavinett et al., 2018; Najafi and Churchland, 2018), usually including stimuli from multiple senses (Angelaki et al., 2009; Chandrasekaran, 2017). The acquired sensory information crucially depends on our actions — what we see, hear, and touch is influenced by our movements — a process known as active sensing (Schroeder et al., 2010; Yang et al., 2016b). For example, imagine attempting to cross the road on a rainy night. You need to interact with the environment, that is, turn your head and move your eyes, and process the incoming stimuli (e.g., car lights, slippery ground) to decide whether and when it is safe to do so. If you feel the road is slippery, you may need to monitor your steps and at the same time you may have to walk faster or step back if a car is approaching.

This example indicates that in real-world settings most perceptual decisions are made during active behaviors (Musall et al., 2019). The quality of the acquired evidence is driven by such active behaviors, which, in turn, affect the efficiency of the perceptual decisions that we make as a result of this active sensing process (Yang et al., 2016a; Gottlieb and Oudeyer, 2018). A first crucial element of fast and accurate perceptual decisions is the combination of evidence from different sensory streams (e.g., sight and touch) to form a unified percept and reduce uncertainty about the stimulus (Ernst and Banks, 2002). However, while there is extensive evidence that the integration of information from different sensory modalities improves perceptual choice accuracy (Lewis and Noppeney, 2010; Raposo et al., 2012) and response time (RT) (Drugowitsch et al., 2014), multisensory information processing has not been studied in an active scenario, where human participants are allowed to implement their own strategy for gathering evidence, as is the case in real-life settings.

Here we addressed this gap in the literature aiming to uncover the neural mechanisms underlying the formation of perceptual decisions via the active acquisition and processing of multisensory information. To achieve this, we capitalized on our previous work probing the neural correlates of active tactile decisions (Delis et al., 2018) and extended it to a multisensory setting that includes visual and haptic information presented simultaneously or separately. We hypothesized that the neural encoding of active sensory experience would be enhanced when multisensory information was available and that this neural multisensory gain would lead to improvements in decision-making performance.

An important aspect of our study is that the participants had full control of the evolution and duration of each trial. In other words, they could choose how much information to sample, where to sample this information from and for how long. Thus, we first aimed to characterize cortical coupling to continuous active sensing and then combined this with a popular sequential-sampling model of decision-making, the drift diffusion model (DDM) (Ratcliff and McKoon, 2008), to understand how the identified representations of active sensing behaviors influence decisions in the human brain. Here, to bridge the gap between active evidence acquisition and decision formation, we used the neural correlates of active (multi-)sensing to constrain the DDM.

Finally, to quantify cross-modal interactions in the brain, we applied a novel information-theoretic framework named partial information decomposition (PID) (Williams and Beer, 2010; Timme et al., 2014; Ince, 2017). PID quantifies the contribution of (1) each sensory modality and (2) cross-modal representational interactions (“redundant” or “synergistic”) to the multisensory neural representation (Park et al., 2018). Redundancy measures the similarity of the neural representation of the two modalities, while synergy indicates a better prediction of the neural response from both modalities simultaneously. Ultimately, this approach revealed the interactions between representations of different sensing modalities in the brain and shed light onto their role in decision-making behavior.

## Materials and Methods

### Experimental design and paradigm

Fourteen healthy right-handed participants (8 female, aged 24 ± 2 years) performed a two-alternative forced choice discrimination task during which they had to compare the amplitudes of two sinusoidal stimuli of the same frequency. All experimental procedures have been reviewed and approved by the Institutional Review Board at Columbia University.

**Figure 1.**
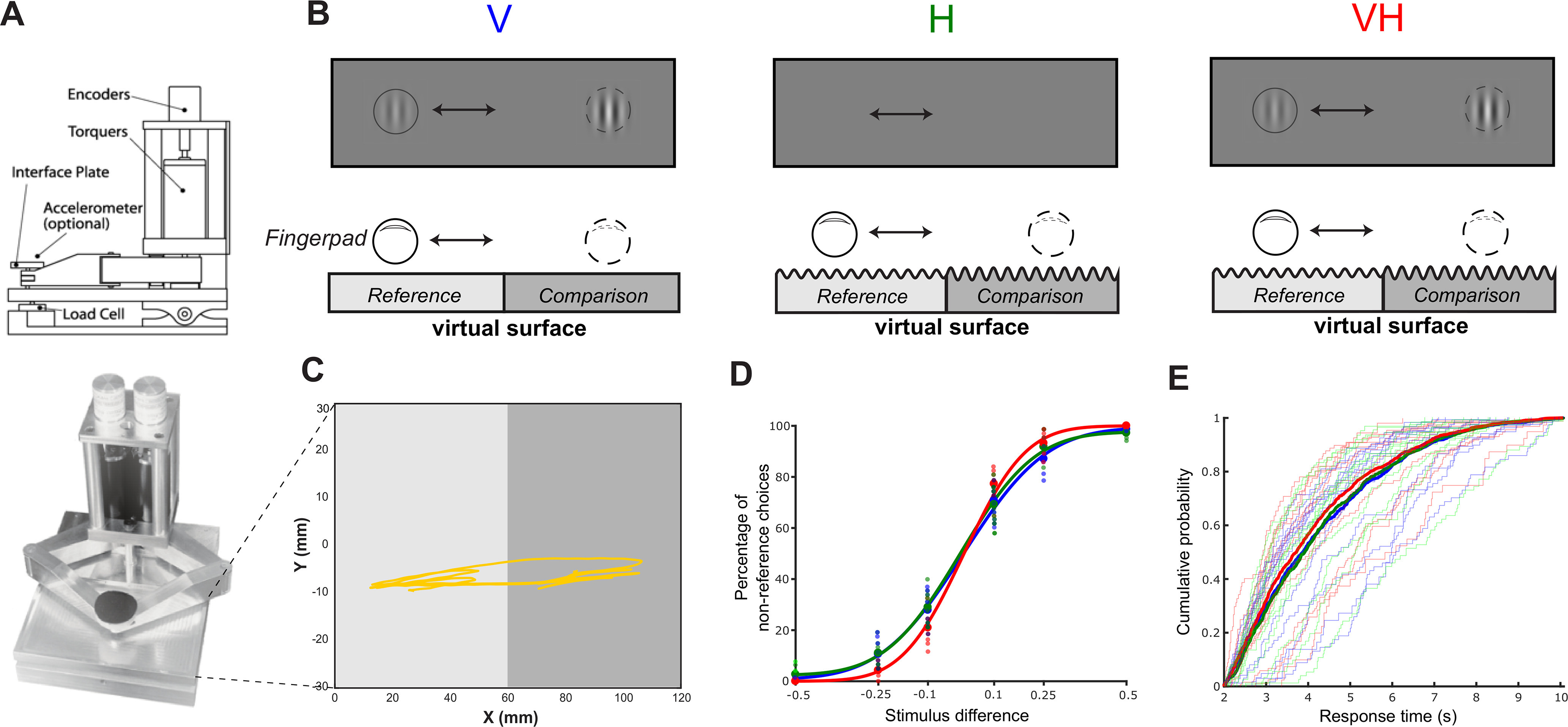
Experimental design and behavioral results. ***A***, The Pantograph is a haptic device used to render virtual surfaces that can be actively sensed. Top, The parts of the Pantograph shown from a lateral view. Participants placed their index finger on the interface plate. Bottom, The Pantograph device used in this experiment. ***B***, The stimulus in the three sensory conditions. We programmed the Pantograph to generate a virtual grating texture. The workspace was split into two subspaces (L and R) that differed in the amplitude of the virtual surface that the participants actively sensed. One of the two sides (randomly assigned) had the reference amplitude (equal to 1), and the other had the comparison amplitude that varied on each trial taking one of the values: 0.5, 0.75, 0.9, 1.1, 1.25, and 1.5. Participants performed the task using V, H, or VH. Amplitude of the stimulus in the haptic domain (H) was translated as contrast in the visual domain (V). Crucially, to match the H condition, only a moving dot following the participant's finger was revealed on the screen in V. ***C***, Index finger trajectory indicating the scanning pattern of the virtual texture in one trial. On this trial, the participant actively sensed the left subspace first, then moved to the right subspace and explored it before coming back to the left subspace again and reporting their choice. ***D***, Psychometric curves indicating the percentage of nonreference choices for all three sensory conditions (blue represents V; green represents H; red represents VH) and for all stimulus differences. Large dots represent average percentage of choices across participants. Smaller dots represent individual participant means. Data are fit using cumulative Gaussian functions. ***E***, Cumulative distributions (CDF) of RTs for all three sensory conditions (blue represents V; green represents H; red represents VH) across all trials of all participants. Thick lines indicate CDFs across all participant data. Thin lines indicate individual participant CDFs for each sensory condition.

To generate visual and tactile stimuli that can be actively sensed, we used a haptic device called a Pantograph (Campion et al., 2005), which can be controlled to generate the sensation of exploring real surfaces (Fig. 1*A*). The Pantograph is a two-dimensional force-feedback device, that is, (1) it produces a 2D tactile output and (2) it simultaneously measures 2D information about the finger position and applied force. Here we used its first property to generate stimulation and the second property to record the kinematics of the movements performed by the participants while they actively explored the presented stimuli. In particular, we split the workspace of the Pantograph (of dimensions 110 mm × 60 mm) into two subspaces (left [L] and right [R], 55 mm × 60 mm each) and generated continuous sinusoidal stimuli of different amplitudes (but same wavelength of 10 mm) in the two subspaces (Fig. 1*B*). Then, we instructed the participants to discriminate the amplitude of the two subspaces as quickly and as accurately as possible (1) using only visual (V) information, (2) using only haptic (H) information, and (3) combining the two sensory cues (VH). Crucially for our investigation here, participants were free to choose how to explore this virtual texture, that is, where and how fast to move their fingers and how long to explore each one of the two sides for before making their perceptual choice. Participants placed their right index finger on the interface plate of the Pantograph (Fig. 1*A*) and moved it freely to explore the textures of both subspaces (Fig. 1*C*) before reporting their choice (i.e., which amplitude is higher) by pressing one of two buttons on a keyboard (left or right arrow) using their left hand.

Specifically, in the H condition, the Pantograph produced sinusoidal forces of different intensity between L and R. When the participants placed their index fingers on the plate (interface) of the Pantograph, these forces at the interface had the effect of causing fingertip deformations and thus tactile sensations that resembled exploring real surfaces. Thus, when moving their finger on the Pantograph, participants had the sensation of touching a rough surface (with different amplitudes between L and R; see Fig. 1*B*, middle). In the V condition, stimuli matching the tactile stimuli were presented on a screen of the same dimensions. More precisely, amplitudes of the sinusoidal virtual texture in H were translated into contrast levels of sinusoidal gratings in V; that is, the participants were seeing black and white stripes of different intensity/contrast between L and R. Presentation of visual stimuli was generated using Psychtoolbox, and visual contrast varied between 0.5 and 1.5 around the default contrast value. The visual angle was 12 ± 6°. Stimulus presentation was controlled by a real-time hardware system (MATLAB XPCTarget) to minimize asynchrony, which was <1 ms. Importantly, to match the sense of touch, only the part of the workspace corresponding to the participant's finger location was revealed on the screen (i.e., a moving dot following the participant's finger; see Fig. 1*B*, left). Thus, in the V condition, grayscale visual textures (of different contrast between L and R) were shown wherever the participants moved their fingers while no forces were applied to the participants' fingers (i.e., no H stimulation). Hence, in both sensory domains, participants could only sense the presented stimulus via active exploration (i.e., finger movements on the *x* axis). Accordingly, in the VH condition, both the visual and haptic textures were congruently presented and sensed by the participants using finger movements (Fig. 1*B*, right). Overall, participants had to decide whether L or R had higher amplitude based on their haptic (in H trials), visual (in V trials), or visuo-haptic (in VH trials) perception of this virtual surface. Participants reported that they perceived the V and H signals as one stimulus in the VH condition.

The amplitude difference between L and R (representing the difficulty of the task) varied from trial to trial. On each trial, participants compared between the reference amplitude 1 (presented either on the left or right subspace) and 1 of 6 other amplitude levels (0.5, 0.75, 0.9, 1.1, 1.25, 1.5). Each trial was initiated by the participant. Trial onset was considered the time point at which horizontal finger velocity exceeded 0. Trial duration was determined by the participant and lasted for the whole period during which the participant made exploratory movements to sense the surface. The trial ended when the participant pressed the < or > key on the keyboard with their left hand to indicate their L or R choice. Each participant performed 20 trials for each amplitude level and for each sensory condition (V, H, VH), resulting in *K* = 20 trials × 6 amplitudes × 3 conditions = 360 trials in total. One participant showed poor behavioral performance (accuracy was not significantly different from chance level), and another participant's EEG recordings were significantly contaminated with eye movement artifacts; thus, data from these 2 participants were removed from any subsequent analyses. We report results from the remaining *N* = 12 participants. We also discarded trials in which participants did not respond within 10 s from trial onset or their RTs were shorter than 0.3 s. This resulted in the rejection of 4.9% of the trials.

### Data recording and preprocessing

During performance of the task, we measured (1) the choice accuracy and RT of participants' responses, (2) movement kinematics (*x*, *y* coordinates of finger position recorded by the Pantograph) at a sampling frequency of 1000 Hz, and (3) EEG signals at 2048 sampling frequency using a Biosemi EEG system (ActiveTwo AD-box, 64 Ag-AgCl active electrodes, 10-10 montage).

To compare accuracies and RTs across sensory conditions, we used two-way ANOVAs with factors condition and stimulus difference followed by Bonferroni-corrected *post hoc t* tests. We also fit psychometric curves to the accuracy data of each participant using a cumulative Gaussian distribution and computed the point of subjective equality (PSE) and slope of the curve at the PSE.

Single-trial movement velocity waveforms were computed using the derivatives of the recorded position. EEG recordings were preprocessed using EEGLab (Delorme and Makeig, 2004) as follows. EEG signals were first downsampled to 1000 Hz to match movement kinematics and dynamics. Then, they were bandpass filtered to 1-50 Hz using a Hamming windowed FIR filter. To isolate the purely neural component of the EEG data, we used the following procedure: we first reduced the dimensionality of the EEG data by reconstituting the data using only the top 32 principal components derived from principal component analysis. Although we record from 64 channels, we expect our recordings to span a considerably lower-dimensional space (as a result of correlations, crosstalk, and common sources); thus, to enhance the ability of independent component analysis to identify truly independent components, we reduce the data dimensions to half using principal component analysis. Thereafter, an independent component analysis decomposition of the data was performed using the Infomax algorithm (Bell and Sejnowski, 1995). We then used an independent component analysis-based artifact removal algorithm called MARA (Winkler et al., 2011) to remove independent components attributed to blinks, horizontal eye movements, muscular activity (EMG), and any loose or highly noisy electrodes. MARA assigned each independent component a probability of being an artifact; we removed components with probabilities >0.5.

### Decoding finger kinematics from EEG signals

To assess the neural encoding of the participants' active sensory experience in the three sensory conditions, we used a multivariate linear regression analysis introduced by Di Liberto et al. (2015) and shown in Equation 1 below. As in our previous work (Delis et al., 2018), we hypothesized that the sensorimotor strategy used by the participant can be represented by the velocity profiles of the participant's exploratory movements, which capture changes of movement direction as well as speed changes. Thus, as kinematic feature representing the active sensing behavior, we used 1 d finger velocity on the *x* axis (capturing L-R finger movements), but also finger position (on the *x* axis) yielded qualitatively very similar results. Finger movement in the *y* axis (which did not provide any sensory information) did not show any significant correlation with the EEG signals and was not considered further. We thus performed a multivariate ridge regression (Crosse et al., 2016) predicting the 1 d finger velocity (on the *x* axis) from the EEG data. Specifically, our decoding analysis aimed to reconstruct the movement velocity from a linear combination of the EEG recordings with time lags ranging between –200 and 400 ms with respect to the instantaneous velocity values. Specifically, we aimed to decode the velocity profile *s*(*t*) of the participants' scanning movements from the simultaneously recorded EEG signals *m*(*i,t*), as follows:

(1) $$\hat{s}(t)≅\sum_{\tau }\sum_{i}g(\tau ,i)m(t+\tau ,i)$$

where $\hat{s}(t)$ is the reconstructed finger velocity and, *g*(*i*, τ) is a filter that integrates information spatially across EEG channels *i* and temporally across time lags τ to decode the velocity profile from the EEG recordings. Here we used τ ∈ [–200 ms, 400 ms]; that is, we examined the EEG information about the finger velocity at time *t* from *t* –200 ms (200 ms earlier) up to *t* 400 ms (400 ms later). Varying these lags did not improve reconstruction performance and yielded qualitatively similar results with the main effects always in the [–200 ms, 400 ms] temporal window, so we used this window for all our further analyses. To learn the decoding filters and compute the velocity approximation accuracy (*r*^2^) between the original and the reconstructed velocity profiles, we used the multivariate temporal response function MATLAB Toolbox implementing regularized linear (ridge) regression (Crosse et al., 2016). In all our filter estimations, we used a cross-validation procedure. We first randomly split our data into two sets: a training set (80% of the trials) to learn the filters and a test set (the remaining 20% of the trials) to apply the filters to and compute the reported *r*^2^ values. In the training set, we performed fivefold cross-validation to identify the optimal value of the ridge parameter λ (varying λ = 2°, …, 2^20^) that maximizes *r*^2^ between the estimated and the measured velocity. These investigations revealed that values of λ between 2° and 2^4^ yielded almost identical *r*^2^ across all models; thus, we used λ = 2^2^ for all models for consistency.

Since the weights of the decoding filters are not interpretable in terms of the neural origins of the underlying processes (Haufe et al., 2014), we transformed them into encoding filters *f*(τ,*i*) using the “forward model” formalism (Parra et al., 2002; Haufe et al., 2014), as follows:

(2) $$f(\tau ,i)=\frac{m{\left(t,i\right)}^{T}m(t,i)g(i,\tau )}{\hat{s}{\left(t\right)}^{T}\hat{s}(t)}$$

We then plotted the weights of the forward models *f*(τ,*i*) at specific time lags τ as scalp maps to visualize the relationship between sensorimotor behavior and neural activity in each one of the three sensory conditions (V, H, VH).

### Statistical analysis of EEG-behavior couplings

To determine statistical significance of the learned EEG-velocity mappings, we randomized the phase spectrum of the EEG signals, which disrupted the temporal relationship between the EEG activity and the kinematics while preserving the autocorrelation structure of the signals (Theiler et al., 1992). We generated 1000 phase-randomized surrogates of the EEG data and computed correlations with the kinematics to define the null distribution from which we estimated *p* values. This phase-randomization procedure maintains the magnitude spectrum of the EEG signals, thus conserving their autocorrelation structure, which is a fundamental feature of the original signals when the significance of cross-correlation is assessed. Hence, using this procedure, the obtained surrogates that define the null distribution are a more plausible comparison (resulting in a stricter statistical test) than randomly shuffled surrogates.

### Informed modeling of decision-making performance

Having characterized the cortical coupling to the sensorimotor strategies in the three sensory conditions, we then probed the relationship between the identified EEG-velocity couplings and decision-making performance. To provide this missing link between active sensing and decision formation, we implemented a hierarchical DDM (HDDM), a well-known cognitive model of decision-making behavior, and informed it with the results of our previous decoding analysis.

We fit the participants' decision-making performance (i.e., accuracy and RT) with an HDDM (Wabersich and Vandekerckhove, 2014), which assumes a stochastic accumulation of sensory evidence over time, toward one of two decision boundaries corresponding to correct and incorrect choices (Ratcliff and McKoon, 2008). The model returns estimates of internal components of processing, such as the rate of evidence accumulation (drift rate), the distance between decision boundaries controlling the amount of evidence required for a decision (decision boundary), a possible bias toward one of the two choices (starting point), and the duration of nondecision processes (nondecision time), which include stimulus encoding and response production. As per common practice, we assumed that stimulus differences affected the drift rate (Palmer et al., 2005).

In short, the model iteratively adjusts the above parameters to maximize the summed log likelihood of the predicted mean RT and accuracy. The DDM parameters were estimated in a hierarchical Bayesian framework, in which prior distributions of the model parameters were updated on the basis of the likelihood of the data given the model, to yield posterior distributions (Wiecki et al., 2013; Wabersich and Vandekerckhove, 2014). The use of Bayesian analysis, and specifically the HDDM, has several benefits relative to traditional DDM analysis. First, this framework supports the use of other variables as regressors of the model parameters to assess relations of the model parameters with other physiological or behavioral data (Frank et al., 2015; Turner et al., 2015; Nunez et al., 2017). This regression model, which is included in HDDM, allows estimation of trial-by-trial influences of a covariate (e.g., a brain measure) onto DDM parameters. In other words, trial-by-trial fluctuations of the estimated HDDM parameters can be approximated as a linear combination of other trial-by-trial measures of cognitive function (Wiecki et al., 2013; Forstmann et al., 2016). This property of the HDDM enabled us to establish the link between the results of the EEG-velocity coupling analysis and the decision parameters of the model, by using the EEG-velocity couplings as predictors of the HDDM parameters, as explained below (for an example of such a linear regression of the drift rate parameter, also see Eq. 3). Second, the model estimates posterior distributions of the main parameters (instead of deterministic values), which directly convey the uncertainty associated with parameter estimates (Kruschke, 2010). Third, as a result of the above, the hierarchical structure of the model allows estimation of the HDDM parameters across participants and conditions, thus yielding distributions at different levels of the model hierarchy (e.g., the population level and the participant level, respectively). In this way, the HDDM capitalizes on the statistical power offered by pooling data across participants (population-level parameters) but at the same time accounts for differences across participants (represented by the variance of the population-level distribution and the individual participant-level estimates). Fourth, the Bayesian hierarchical framework has been shown to be especially effective when the number of observations is low (Ratcliff and Childers, 2015).

To implement the hierarchical DDM, we used the JAGS Wiener module (Wabersich and Vandekerckhove, 2014) in JAGS (Plummer, 2003), via the Matjags interface in MATLAB to estimate posterior distributions. For each trial, the likelihood of accuracy and RT was assessed by providing the Wiener first-passage time distribution with the four model parameters (boundary separation, starting point, nondecision time, and drift rate). Capitalizing on the advantages of HDDM, we ran the model pooling data across all participants and conditions and estimated both population-level and participant-level distributions. Parameters were drawn from uniformly distributed priors and were estimated with noninformative mean and SD group priors. As per standard practice for accuracy-coded data, the starting point was set as the midpoint between the two decision boundaries as participants could not develop a bias toward correct or incorrect choices. For each model, we ran three separate Markov chains with 5500 samples of the posterior parameters each; the first 500 were discarded (as “burn-in”) and the rest were subsampled (“thinned”) by a factor of 50 following the conventional approach to MCMC sampling whereby initial samples are likely to be unreliable because of the selection of a random starting point and neighboring samples are likely to be highly correlated (Wabersich and Vandekerckhove, 2014). The remaining samples constituted the probability distributions of each estimated parameter. To ensure convergence of the chains, we computed the Gelman-Rubin *R*^2^ statistic (which compares within-chain and between-chain variance) and verified that all group-level parameters had an R2 close to 1 and always <1.01.

Here, to obtain a mechanistic account of the effect of EEG-velocity coupling on decision-making behavior, we incorporated the single-trial measures of these couplings (*r*^2^ values defined above) into the HDDM parameter estimation (see Fig. 3*B*). Specifically, as part of the model fitting within the HDDM framework, we used the single-trial velocity reconstruction accuracies *r*^2^ as regressors of the decision parameters to assess the relationship between trial-to-trial variations in EEG-velocity couplings and each model parameter. Furthermore, to characterize the effect of active sensing movements on decision formation, we also incorporated movement parameters in the HDDM framework. Specifically, we computed the following movement parameters: (1) the average finger velocity (*v_m_*) on each trial; (2) the number of crossings (*n_cr_*) between L and R, which is an indicator of the time it took participants to switch between the two stimuli; and (3) the time participants spent exploring one of the two stimuli (here we arbitrarily selected the low-amplitude stimulus on each trial, *t_low_*) as an indicator of exploration time. To understand how these movement parameters affect the decision-making process and specifically whether they relate to (1) sensory processing and movement planning/execution (i.e., nondecision processes) and/or (2) evidence accumulation (i.e., decision processes) and/or (3) the speed-accuracy trade-off adopted by the participants, we used these parameters as regressors for nondecision time, drift rate, and decision boundary, as follows:

(3) $$\tau =\beta_{0}+\beta_{1}*r^{2}+\beta_{v}*v_{m}+\beta_{sw}*n_{cr}+\beta_{\exp}*t_{low}$$

(4) $$\delta =\gamma_{0}+\gamma_{1}*r^{2}*s+\gamma_{sw}*n_{cr}+\gamma_{\exp}*t_{low}$$

(5) $$\alpha =\theta_{0}+\vartheta_{1}*r^{2}+\vartheta_{v}*v_{m}+\vartheta_{sw}*n_{cr}+\vartheta_{\exp}*t_{low}$$

where τ, δ, and α represent the single-trial nondecision time, drift rate, and decision boundary, respectively. Velocity reconstruction accuracy *r^2^*, mean finger velocity *v_m_*, number of crossings *n_cr_*, and time spent exploring the lower-amplitude stimulus *t_low_* are the single-trial predictor variables with regression coefficients β*_i_*, γ*_i_*, and δ*_i_*, respectively, and *s* = 0.1, 0.25, 0.5 is the stimulus difference on each trial *k* = 1,…,*K* of each participant *n* = 1,…, *N*. As per common practice, we modeled a linear relationship between drift rates and stimulus differences reflecting the dependence of the speed of information integration on the amount of evidence available (Palmer et al., 2005; Ratcliff and McKoon, 2008).

By using the above regression approach, we were able to test the influence of the above EEG and movement parameters on each of the HDDM parameters. Thus, we tested different models in which the single-trial values of the above parameters were used as predictors for all combinations of the HDDM parameters (drift rate, nondecision time, and decision boundary). To select the best-fitting model, we used the deviance information criterion (DIC), a measure widely used for fit assessment and comparison of hierarchical models (Spiegelhalter et al., 2002). DIC selects the model that achieves the best trade-off between goodness-of-fit and model complexity. Lower DIC values favor models with the highest likelihood and least degrees of freedom.

### Statistical analysis of modeling results

Posterior probability densities of each regression coefficient were estimated using the sampling procedure described above. Significantly positive (negative) effects were determined when >95% of the posterior density was higher (lower) than 0. To take into account the hierarchical structure of the model which estimated both population-level distributions and participant-level distributions of the parameters, all statistical tests at the population level were performed by contrasting the group-level distributions (not the individual participant means) across sensory conditions. This hierarchical statistical testing has been shown to reduce biases and actually yield conservative effect sizes (Boehm et al., 2018).

### PID

We then aimed to uncover whether the visual (V) and haptic (H) neural representations of active sensing contained the same information (redundancy) that is present in the multisensory (VH) representation or to what extent their contributions are distinct (unique information) or complementary (synergy). To achieve this, we used the PID (Williams and Beer, 2010; Timme et al., 2014) applied to the predictions of the finger velocity encoding models learned in the different experimental conditions. PID provides an information theoretic approach to compare the outputs of different predictive models that goes beyond simply comparing accuracy to determine whether the different models share or convey unique predictive information content (Daube et al., 2019b). PID extends the concept of co-information (McGill, 1954), which is defined as follows:

(6) I(VH;V;H)=I(VH;V) + I(VH;H)−I(VH;[V,H])

where *I*(*X;Y*) denotes the mutual information (MI) between variables *X* and *Y*. MI is a nonparametric measure of dependence between two variables which has the unique property that its effect size is additive (Shannon, 1948). Hence, co-information (also called interaction information when defined with opposite sign) quantifies the difference between the sum of the MI when each modality is considered alone and the MI when the two modalities are observed together (Park et al., 2018).

Positive values of this difference indicate that some information about the predictions of the multisensory VH model is shared between the predictions obtained from the models trained in the unisensory V and H conditions (i.e., there are common or redundant representations of finger velocity in both V and H conditions). Negative values of the interaction information indicate a super-additive or synergistic interaction between the predictions of the V and H models; that is, the two models provide more information about the multisensory (VH) prediction when observed together than would be expected from observing each individually. However, interaction information measures the net difference between synergy and redundancy in the system; thus, it is possible to have zero interaction information, even in the presence of redundant and synergistic interactions that cancel out in the net value (Williams and Beer, 2010; Ince, 2017). This occurs because classic Shannon quantities cannot separate redundant and synergistic contributions, which has led to a growing field developing PID measures to address this shortcoming.

To give a simple example of such a case, let us consider three variables, each consisting of two bits (i.e., binary (0/1) variables with p(0) = p(1) = 0.5). Let us also assume that the first bit is shared between all three variables and the second bit follows the XOR distribution across the three variables. In this case, there is clear redundancy and synergistic structure, but co-information/interaction information is zero (Griffith and Koch, 2014).

More precisely, PID addresses this methodological problem by decomposing MI into unique redundant and synergistic components, as follows:

(7) $$I(VH;V;H)=I_{uni}(VH;V)+I_{uni}(VH;H)+I_{red}(VH;V,H)+I_{syn}(VH;V,H)$$

where *I_uni_*(*VH;V*) is the part of the VH model predictions that can be explained only from the V model predictions, *I_uni_(VH;H)* is the part of the VH model predictions that can be explained only from the H model predictions, *I_red_*(*VH;V,H*) is the part of the VH model predictions that is common (redundant) to both the V and H model predictions, and *I_syn_*(*VH;V,H*) is the extra (synergistic) information about the VH model predictions that arises when both V and H predictions are considered together. PID decomposes the joint MI between two predictor signals (here the EEG activity predicted from an encoding model trained in the unisensory V, H conditions) and a target signal (here the EEG activity predicted from an encoding model trained in the multisensory VH condition) into four terms: redundancy, the unique information in each predictor, and synergy. Redundancy quantifies the information in the target signal that is shared between the two predictor signals. Synergy quantifies improvement in prediction of the target when both predictors are observed together and represents information about the target signal which cannot be obtained from the individual predictors separately.

To perform PID here, we used a recent implementation based on common change in surprisal for Gaussian variables (Ince, 2017), which has been shown to be effective when applied to neuroimaging data (Park et al., 2018; Daube et al., 2019a).

To implement the above approach on our data, we used the recordings of the VH condition where the two unisensory representations of active sensory experience could be directly compared with the multisensory representation. We took the velocity-encoding models obtained in each condition (V, H, VH) and applied them to the VH data (see Eq. 3) to obtain the V, H, and VH predictions of each EEG sensor activity for all VH trials. Since the unisensory models (V, H) were fit in the corresponding unisensory condition, they could only have learned a unisensory representation, whereas the VH model learned a multisensory representation of active sensing velocity. Thus, we applied PID for each participant separately to predict the VH model predictions from the two unisensory V and H model predictions, which enabled us to quantify the cross-modal interactions between the two unisensory representations across all EEG sensors.

### Statistical analysis of PID results

We performed this decomposition independently for each EEG channel and obtained scalp maps for the four PID terms (redundant information, unique information of A, unique information of V, synergistic information) for each participant. To avoid overfitting, we implemented a fivefold cross-validation procedure. We randomly split the VH data into five subsets used four of them to learn the VH, V, and H models and the held-out set to perform the PID on. We repeated this process 5 times to obtain PID values for all the VH data. To assess statistical significance of the obtained values, we performed a permutation test. Specifically, we shuffled the target signal (i.e., the VH model of active sensing) 1000 times while keeping the two predictor signals (V and H models, respectively) unchanged and applied PID to predict the VH model surrogate data. Output values of the original PID decomposition were considered significant if they exceeded the 99th percentile of the distribution of the surrogate data. Multiple comparisons were corrected for using FDR (Genovese et al., 2002).

## Results

We collected behavioral and EEG data while 14 participants actively interrogated a two-dimensional texture stimulus that differed in its amplitude in one dimension (L vs R). Participants used V, H, or VH to make a 2-alternative forced perceptual choice, that is, report (via a key press) as quickly and as accurately as possible on which side (L or R) the texture stimulus had higher amplitude (Fig. 1*B*). To sample information from both sides, participants performed finger movements scanning the workspace of the Pantograph before reaching a decision (Fig. 1*C*).

In the H condition, the Pantograph (for more details on the device used to generate the stimuli, see Materials and Methods) was programmed to produce sinusoidal forces, which yielded the sensation of exploring a rough texture surface (with different amplitudes between L and R, when participants moved their index finger on the workspace of the Pantograph; see Fig. 1*B*, middle). In the visual domain, participants were moving their fingers to reveal grayscale stripes of different intensity/contrast between L and R (Fig. 1*B*, left). In the VH condition, both the visual and haptic textures were congruently presented wherever the participants moved their fingers (Fig. 1*B*, right). Overall, participants had to decide whether L or R had higher amplitude based on their haptic (in H trials), visual (in V trials), or visuo-haptic (in VH trials) perception of this virtual surface.

### Multisensory gain in behavioral performance

Multisensory stimulation resulted in significantly higher discrimination accuracy (91.5 ± 2.1% in VH vs 85.8 ± 2.2% in V and 86.3 ± 2.2% in H, two-way ANOVA with factors condition and stimulus difference, *F*_(2,99)_ = 5.64, *p* < 0.005, see also slopes in the corresponding psychometric curves in Fig. 1*D*, *PSE_v_* = 0.034 ± 0.013, *PSE_h_* = −0.001 ± 0.009 *PSE_vh_* = −0.019 ± 0.007, *slope_v_* = 2.397 ± 0.2964, *slope_h_* = 1.826 ± 0.147, *slope_vh_* = 3.001 ± 0.2514) compared with the unisensory conditions (*post hoc t* tests, Bonferroni-corrected, *p* = 0.009 for V-VH and *p* = 0.019 for H-VH). RTs also reduced in VH (4.11 ± 0.30 s vs 4.41 ± 0.31 s in V and 4.25 ± 0.29 s in H, two-way ANOVA with factors condition and stimulus difference, *F*_(2,99)_ = 3.19, *p* = 0.045, see also corresponding cumulative distribution functions in the three conditions, Fig. 1*E*). This result was significant at the population level for VH versus V differences (*post hoc* t test, *p* = 0.021, Bonferroni-corrected) but not VH versus H differences (*post hoc t* test, *p* = 0.066, Bonferroni-corrected) in RTs. As expected, we also found a main effect of stimulus differences, with accuracy increasing (*F*_(2)_ = 91.82, *p* < 0.0001) and reaction times decreasing (*F*_(2)_ = 4.56, *p* < 0.02) with larger stimulus differences, respectively. There was no interaction between the sensory condition and stimulus difference on either measure (accuracy: *F*_(4)_ = 0.66, *p* = 0.62; reaction times: *F*_(4)_ = 0.05, *p* = 0.99). Together, these results indicate that multisensory information increased decision-making performance.

### Reconstruction of active sensing velocity from EEG recordings

We then aimed to establish a relationship between brain activity and the active sensory experience of the participants in each one of the three sensory conditions. To this end, we performed a multivariate ridge regression (Crosse et al., 2016) between the EEG data and the 1 d finger velocity data (on the *x* axis) to quantify neural encoding of sensorimotor behavior.

**Figure 2.**
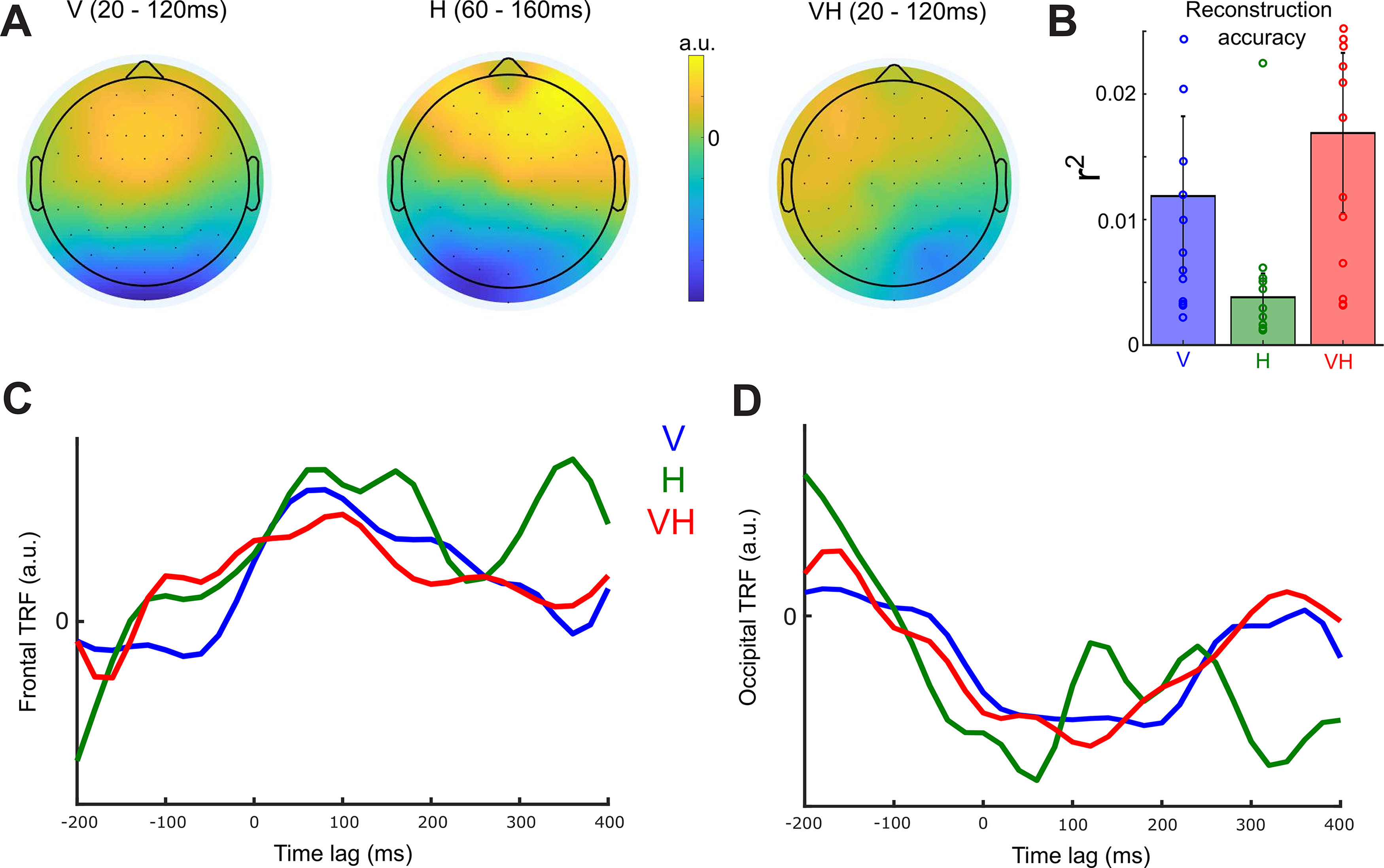
Results of velocity reconstruction analysis using EEG signals. ***A***, Scalp topographies of the forward models representing neural encoding of instantaneous finger velocity for the three sensory conditions. The presented scalp maps show velocity-encoding EEG signals averaged over the following time windows: 20 and 120 ms lags between velocity and EEG for V and VH, and 60 and 160 ms lags for H. ***B***, Accuracy of the velocity reconstruction from the EEG signals measured using the squared correlation coefficient (*r*^2^) between the original and the approximated velocity profile in the three sensory conditions (blue represents V; green represents H; red represents VH). Bars represent means across participants. Error bars indicate SEM. Dots represent individual participant data. ***C***, ***D***, Temporal response functions (TRFs) of the velocity-encoding EEG activity in the three sensory conditions (blue represents V; green represents H; red represents VH) averaged over frontal electrodes (in ***C***) and over occipital electrodes (in ***D***).

This analysis yielded the optimal linear combination of EEG channel activations with time lags ranging between –200 and 400 ms that approximated the measured movement velocities. We found that reconstruction accuracy *r*^2^ was above chance level in all sensory conditions (all *p* values < 0.01; Fig. 2*B*). To obtain interpretable topographies of the neural activity underlying these EEG-velocity couplings, we inverted the obtained velocity-decoding (backward) models into velocity-encoding (forward) models (Parra et al., 2005; Haufe et al., 2014). This revealed that centro-frontal locations (with positive weights) and occipital locations (with negative weights) contributed most to velocity reconstruction in the three sensory conditions with time lags ranging from 20 to 160 ms; Figure 2*A* shows the scalp topographies of the forward models, and Figure 2*C*, *D* shows the corresponding temporal response functions (averaged across frontal and occipital channels, respectively) in the three sensory conditions.

### Impact of active multi-sensing on the quality of perceptual evidence

To characterize the relationship between the identified EEG-velocity couplings and decision-making performance, we used an HDDM. In brief, the HDDM decomposes task performance (i.e., accuracy and RT), into internal components of processing representing the rate of evidence integration (drift rate, δ), the amount of evidence required to make a choice (decision boundary separation, α), and the duration of other processes, such as stimulus encoding and response production (nondecision time, τ). Ultimately, by comparing the obtained values of all three core HDDM parameters across the V, H, and VH trials, we could associate any behavioral differences resulting from the deployment of multisensory information (more accurate and faster perceptual choices as in Fig. 1) to the constituent internal process reflected by each model parameter.

**Figure 3.**
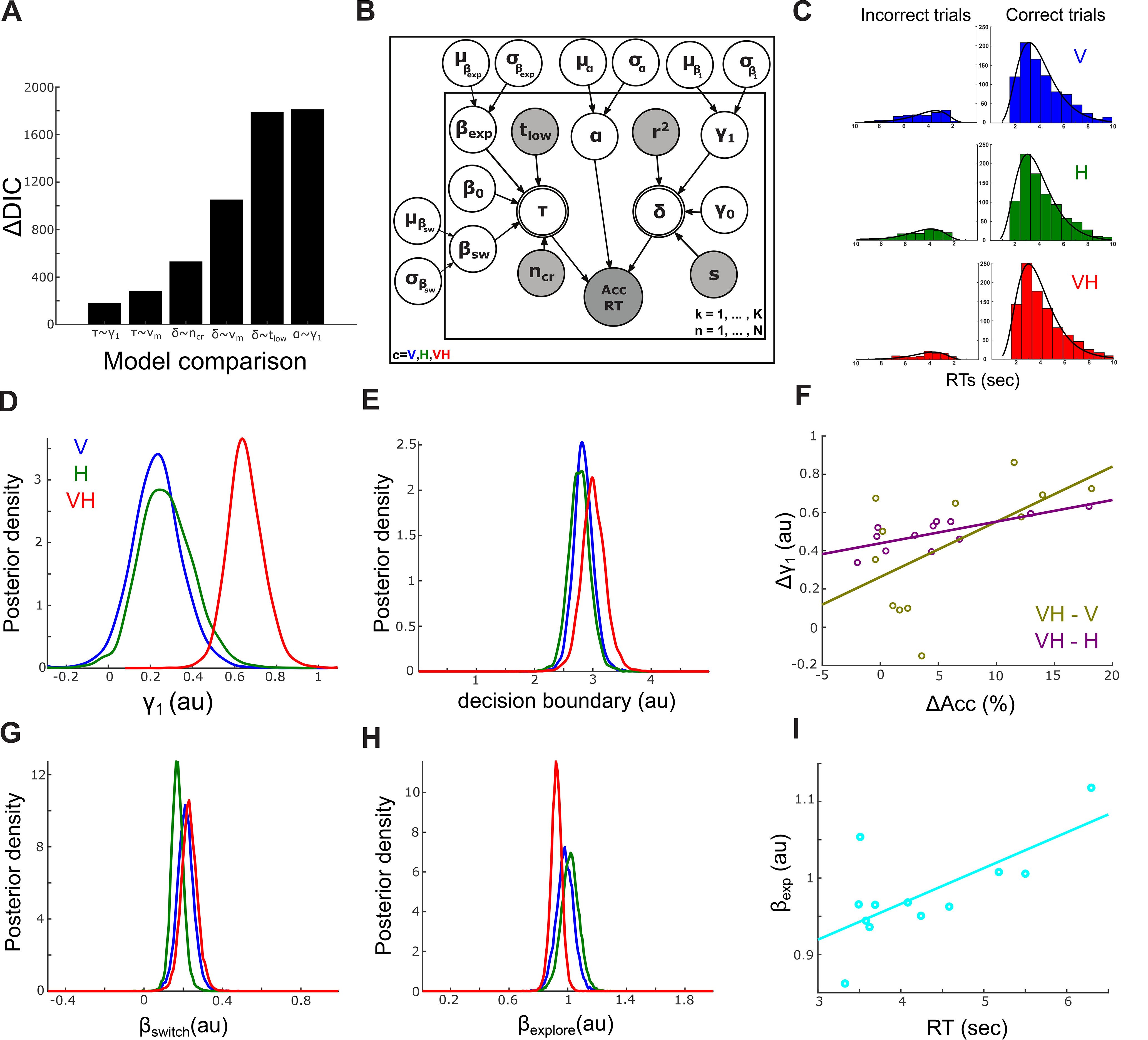
Informed modeling of decision-making behavior. ***A***, Comparison of the best-fitting model (with *r*^2^ as a regressor of drift rate δ only and *n_cr_*, *t_low_* as regressors of nondecision time τ only) with alternate models using the DIC. Positive ΔDIC (DIC_model_ – DIC_optimal_) values for all six models indicate that the model of choice achieved a better trade-off between goodness of fit and number of free parameters. ***B***, Graphical representation showing hierarchical estimation of HDDM parameters. Round nodes represent continuous random variables. Double-bordered nodes represent variables defined in terms of other variables. Shaded nodes represent recorded or computed signals, that is, single-trial behavioral data (accuracy, RT, and stimulus differences, *s*), EEG-velocity couplings (*r^2^*), and kinematic parameters (*n_cr_*, *t_low_*). Parameters are modeled as Gaussian random variables with inferred means μ and variances σ^2^. Plates denote that multiple random variables share the same parents and children. The outer plate is over sensory conditions (V, H, VH), and the inner plate is over all trials (*K*) and participants (*N*). ***C***, Behavioral RT distributions are shown as histograms for each sensory condition (blue represents V; green represents H; red represents VH) for correct (right) and incorrect (left) trials together with the HDDM fits (black lines). Higher histogram values on the right indicate higher proportion of correct choices. ***D***, Posterior distributions of regression coefficients (γ_1_) of the EEG-velocity couplings (*r*^2^), as predictors of the drift rate (δ) of the HDDM shown in ***A***. The three colored curves indicate posterior distributions for the three sensory conditions (blue represents V; green represents H; red represents VH). ***E***, Posterior distributions of decision boundaries for the three sensory conditions (blue represents V; green represents H; red represents VH). ***F***, Cross-participant correlation of differences in choice accuracy (ΔAcc, *x* axis) and differences in β_1_ (Δβ_1_, *y* axis) between the multisensory (VH) and the two unisensory (V, H) conditions (yellow represents VH-V; purple represents VH-H). ***G***, Posterior distributions of regression coefficients (β_sw_) of the number of crossings between L and R (n_cr_), as predictor of nondecision time (τ) of the HDDM shown in ***A***. ***H***, Posterior distributions of regression coefficients (β_exp_) of the time spent on the low-amplitude stimulus (t_low_), as predictor of nondecision time (τ) of the HDDM shown in ***A***. ***I***, Cross-participant correlation of average RTs across trials and sensory conditions (*x* axis) and β_exp_ (*y* axis).

Here, to obtain a mechanistic account of the formation of perceptual decisions via the active sampling of (multi-)sensory information, we incorporated the single-trial measures of brain-sensing couplings (*r*^2^ values) into the HDDM parameter estimation (Fig. 3*B*). Specifically, we applied the obtained decoding filters to the single-trial EEG data and computed velocity reconstruction accuracies for each trial of each sensory condition (using a nested cross-validation process; for more details, see Materials and Methods). Then, as part of the HDDM fitting process, we integrated these single-trial *r*^2^ values in the HDDM framework by using them as regressors of the three core HDDM parameters (drift rate, nondecision time, and decision boundary; see Materials and Methods). The corresponding regression coefficients were estimated together with the HDDM parameters, thus enabling the assessment of the relationship between trial-to-trial variations in EEG-velocity couplings and each model parameter. We also used as regressors three movement parameters (average velocity *v_m_*, number of crossings between L and R *n_cr_*, and time spent on the lower amplitude stimulus *t_low_*), which served to dissociate the effect of the exploratory movements (captured by these parameters) on decision formation from the effect of the neural encoding of these active sensing movements (captured by *r*^2^).

We found that the best-fitting model (achieving the best complexity-approximation trade-off as evaluated by the DIC; Fig. 3*A*) was the one using *r*^2^ as regressor of the drift rate only and *n_cr_, t_low_* as regressors of nondecision time only (Fig. 3*B* shows a graphical illustration of the best-fitting model, and Fig. 3*C* shows the model fitting of the accuracy and RT data where bars represent actual data and lines represent model fits). The means and CIs of the estimated values of the three core HDDM parameters are reported in Table 1. Crucially for our investigation here, the EEG-velocity couplings *r*^2^ were predictive of drift rates in single trials (regression coefficients β_1_ were larger than zero for all three sensory conditions, *Prob*(γ_1_ (*V*) > 0) > 0.97, *Prob*(γ_1_ (*H*) > 0) > 0.99, *Prob*(γ_1_ (*VH*) > 0) > 0.999; Fig. 3*D*). Furthermore, the contribution of *r*^2^ to drift rate was higher in VH trials compared with V and H trials (*Prob*(γ_1_ (*VH*) > γ_1_ (*V*)) > 0.95 and *Prob*(γ_1_ (*VH*) > γ_1_ (*H*)) > 0.99; Fig. 3*D*), indicating a multisensory enhancement of evidence accumulation rates via an increased weighting of the EEG-velocity couplings in the VH condition.

**Table 1.** Estimated values of the three core HDDM parameters for the best-fitting model

Parameter	Mean	CI (5%)	CI (95%)
Drift rate (δ)	0.897	0.628	1.162
Nondecision time (τ)	2.897	2.710	3.045
Decision boundary (α)	2.853	2.501	3.256

We then examined whether this multisensory gain could explain the observed improvements in behavioral performance when multisensory information is available. Indeed, this enhanced contribution of *r*^2^ to drift rate was predictive of multisensory improvements in behavioral performance. Specifically, cross-participant differences in γ_1_'s across conditions correlated with the reported increases in accuracy (*r* = 0.58, *p* = 0.049 for VH vs V and *r* = 0.75, *p* = 0.005 for VH vs H; Fig. 3*F*), suggesting that differences in accuracies across participants were accounted for by the contributions of EEG-velocity couplings to evidence accumulation. Thus, participants with greater drift rate amplification achieved stronger enhancements in their behavioral performance as a result of multisensory information available.

We also found that both switching time between the two stimuli as captured by *n_cr_* and exploration time spent on one of the two stimuli as captured by *t_low_* were predictive of nondecision time (*Prob*(β*_sw_* > 0) > 0.999, *Prob*(β*_exp_* > 0) > 0.999 for all V, H, VH; Fig. 3*G*,*H*) in single trials, indicating that nondecision processes (i.e., related to sensory processing and movement planning/execution) are dependent on switching and exploration times. There was a positive cross-participant correlation (*r* = 0.695, *p* = 0.0121) between β*_exp_* and RT (averaged across trials and sensory conditions), suggesting that participants with larger contributions of exploration time to their nondecision times took longer to respond (Fig. 3*I*). However, we found no reliable difference in the corresponding regression coefficients (β*_sw_*, β*_exp_*) between the three sensory conditions (*Prob*(β*_sw_* (*VH*) > β*_sw_* (*V*)) = 0.632, *Prob*(β*_sw_* (*VH*) > β*_sw_* (*H*)) = 0.843, *Prob*(β*_exp_* (*VH*) > β*_exp_* (*V*)) = 0.107, *Prob*(β*_exp_* (*VH*) > β*_exp_* (*H*)) = 0.210; Fig. 3*G*,*H*). There was also no difference in the decision boundaries in the three sensory conditions (*Prob*(α(*VH*) > α(*V*)) = 0.731, *Prob*(α(*VH*) > α(*H*)) > 0.804; Fig. 3*E*). These results indicate that neither the switching and exploration times nor the amount of evidence required to make a decision was dependent on the sensory condition.

### Quantifying multisensory interactions

**Figure 4.**
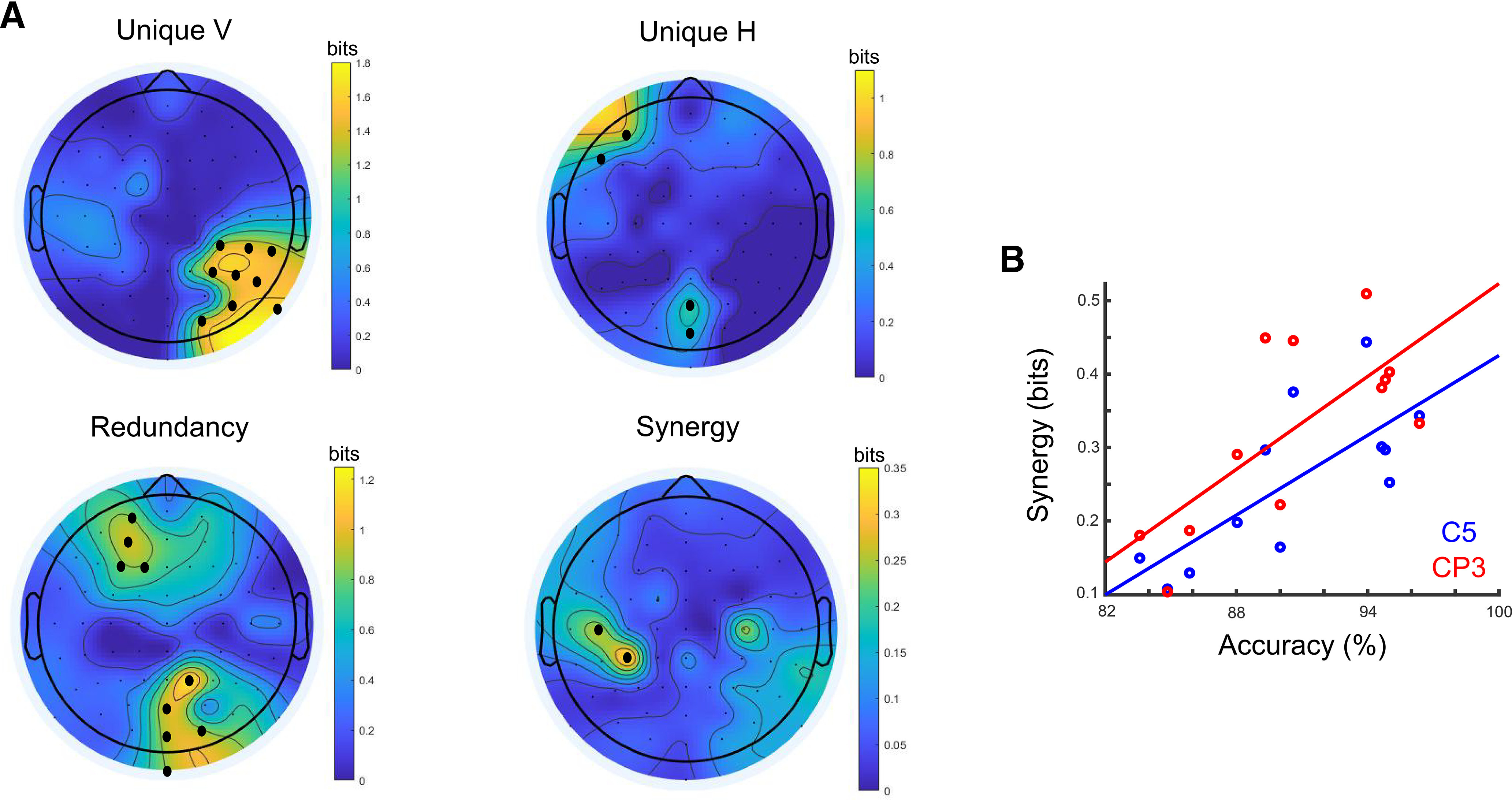
Neural representations and cross-modal interactions. ***A***, Results of PID applied to predict the multisensory (VH) model of active sensing from the two unisensory (V and H) models. Dots on the scalp topographies indicate the EEG channels that provide significant (*p* < 0.01, FDR-corrected) visual unique (top left), haptic unique (top right), redundant (bottom left), and synergistic (bottom right) neural information, respectively. ***B***, Across-subject correlation between synergy in the two significant EEG channels (red represents CP3; blue represents C5) and choice accuracy in the VH condition.

Having established that the neural encoding of the behavioral kinematics is related to the multisensory gain in decision evidence, we then aimed to assess how the neural representations of the two unisensory stimuli (V, H) interact to form a multisensory representation. To this end, we used PID, which enables the quantification of cross-modal representational interactions in the human brain (for details, see Materials and Methods). Specifically, the PID information theoretic framework quantifies the degree to which (1) each unisensory (V,H) representation contributes uniquely to the encoding of active sensing behavior (unique V or H information), (2) the two unisensory (V,H) representations share information about active sensing (redundancy), and (3) the two unisensory (V,H) representations convey more information when observed simultaneously (synergy). Here, we used PID to predict the forward (velocity-encoding) VH model (target signal) from the two unisensory forward models V and H (predictor signals). The decomposition revealed that the V model provided unique information in right parieto-temporal locations, whereas the H model contributed uniquely in left prefrontal and parieto-occipital locations (Fig. 4*A*; all *p* values < 0.01, FDR-corrected). Crucially, we also found multisensory interactions in the form of (1) redundant effects in left prefrontal and parieto-occipital electrodes and (2) synergistic effects over left centroparietal scalp (Fig. 4*A*; all *p* values < 0.01, FDR-corrected). Here, a redundant interaction means that the representation of velocity is common to both the V and H modalities (Ince et al., 2017; Park et al., 2018). A synergistic interaction means a better prediction of the modeled multisensory response can be made when considering both the V and the H representations together (rather than independently). That is, knowledge of the simultaneous combination of the EEG signal predicted by V and H models gives more information about the VH EEG signal.

### Multisensory accuracy scales with synergistic interactions

Next, we investigated the behavioral relevance of the identified cross-modal interactions. In particular, we asked whether the identified synergistic representation of the two modalities was predictive of behavioral performance across participants. Indeed, we found a significant positive correlation (Pearson's *R* = 0.75 and 0.72, all *p* < 0.01) between synergy in both significant channels (CP3 and C5) and accuracy in VH, suggesting that participants with more synergistic representations at left centroparietal electrodes achieved better multisensory performance (Fig. 4*B*). This result suggests that synergy in contralateral centroparietal EEG signals modulates multisensory decision-making behavior. Because of small sample size, we cannot be sure this finding will generalize, but nonetheless report it as an interesting exploratory finding.

## Discussion

In this work, we coupled neural decoding of continuous sensorimotor behavior with modeling of decision-making performance and a quantitative assessment of cross-modal neural interactions to understand how the human brain forms perceptual decisions via the active acquisition of multisensory evidence. We showed that the neural encoding of active sensing modulates the decision evidence regardless of the sensing modality. We further demonstrated that the simultaneous sensing of different modalities enhances this neural coupling and this enhancement drives the dynamics of active multisensory decisions. We finally dissected the neural information conveyed by cross-modal interactions and identified a potential neural mechanism supporting multisensory decisions.

Recent research on active sensing uncovered the strategies implemented by humans to sample sensory information (Yang et al., 2016b). Here we investigated this active sensing approach in a decision-making task using a computational approach that decodes the neural activity that encodes movement kinematics. Crucially, we made a first step in broadening this line of research to (1) include sensory information from multiple modalities and (2) reveal its neural underpinnings. These two developments enabled us to uncover the different sensory representations of active sampling behavior in the human brain.

To achieve this, we implemented an informed cognitive modeling approach that linked the neural correlates and the movement characteristics of active sensing behavior with the cognitive processes involved in decision-making. Specifically, we asked whether decision-making depends on the neural representations of active (multi-)sensing. To answer this question, we used a single-trial measure of the neural encoding of active sensing behavior as predictor of decision-making performance and found that, indeed, trial-to-trial fluctuations of the neural representations of active sensing are predictive of the rate of evidence accumulation for all three sensory conditions (V, H, VH). Crucially, we showed that the multisensory (VH) representation of active sensing was a stronger predictor of drift rate (Fig. 3*D*), thus offering a neural link between active multi-sensing and perceptual decision-making. We also split the motion profile into its two main components: (1) switching between the two alternative stimuli and (2) exploration within one particular stimulus and demonstrated that both components were predictive of the duration of nondecision processes (Fig. 3*G*,*H*), thus simply reflecting the time spent for movement planning and execution and the consequent acquisition and encoding of sensory information. These novel findings were only made possible by the use of an active multi-sensing paradigm in a decision-making task and the joint cognitive modeling of behavioral, neural, and sensorimotor signals.

We then capitalized on the identified neural representations of active (multi-sensing), to dissect cross-modal interactions in the human brain. To this end, we used PID, a recently developed rigorous methodology for the quantification of information conveyed uniquely or jointly by different neural representations (Williams and Beer, 2010; Timme et al., 2014; Ince, 2017). PID further distinguishes between two types of interactions between the neural representations of the two sensory modalities (V, H). A synergistic interaction indicates that a better prediction of the multisensory neural response can be made when the predicted values of the unimodal forward models for V and H are considered jointly rather than independently. Our results suggest that this synergistic interaction of the two neural representations correlates with multisensory behavioral performance (Fig. 4*B*). Instead, a redundant interaction indicates that the two unimodal models provide the same information about the multisensory condition; thus, the multisensory response there is common to both modalities (Park et al., 2018; Daube et al., 2019a). This suggests that the underlying neural signals reflect a modality-invariant representation.

As a result of this analysis, we were able to identify neural signals representing these two types of interactions. Specifically, we found that EEG channels in (parieto-)occipital and prefrontal areas carried redundant representations of the two sensory streams, perhaps reflecting supramodal coding mechanisms of active sensing (Fig. 4*A*, redundancy). This finding is in line with previous research assigning a multimodal role to occipital cortex (Lacey et al., 2007; Murray et al., 2016) and suggesting that multisensory enhancements originate from the sensory cortices (Kayser and Logothetis, 2007; Lakatos et al., 2007; Lewis and Noppeney, 2010). Specifically, recent research involved the visual cortex in audiovisual interactions (Mishra et al., 2007; Cao et al., 2019; Rohe et al., 2019) as well as tactile perception and visuo-haptic interactions (Lucan et al., 2010; Sathian, 2016; Gaglianese et al., 2020). In agreement with the above, here we also found unique H information in parieto-occipital electrodes. Concerning the PFC, recent evidence assigned to it a modality-general role in arbitrating between segregation or fusion of sensory evidence from different modalities (Cao et al., 2019). Thus, the involvement of the PFC in the regulation of adaptive multisensory behaviors in general (Koechlin and Summerfield, 2007; Donoso et al., 2014; Tomov et al., 2018) and perceptual decisions in particular (Heekeren et al., 2006; Philiastides et al., 2011; Rahnev et al., 2016; Sterzer, 2016) makes it a likely contributor to the formation of the most appropriate sensory representation that drives decision-making behavior. In other words, the PFC may support a mechanism gauging candidate (multisensory or unisensory) representations for selecting among multiple strategies to solve the task at hand (Calvert, 2001; Hein et al., 2007; Noppeney et al., 2010; Cao et al., 2019). Our active multi-sensing task requires participants to continuously weigh different sensing strategies and refine their scanning patterns to maximize information gain. Hence, the PFC may capitalize on multisensory information (when of benefit) to support such flexible behavior striking a balance between sampling more evidence and committing to a choice.

The above findings are consistent with our previous study focusing on the tactile modality, which attributed a sensory processing function to occipital cortex (specifically localized to the lateral occipital complex) and a decision formation function to right PFC (middle frontal gyrus) (Delis et al., 2018). Together with the current results, our findings suggest these two brain areas may play a cross-modal role in supporting active perception and decision-making. Overall, our work adds to the existing literature on multisensory interactions by quantifying how sensory representations interact to encode active sensing behaviors.

More importantly, here we revealed a novel functional role for contralateral centroparietal signals in active visuo-haptic decisions. We found that brain signals over left centroparietal scalp locations showed stronger encoding of active sensing when the two sensory streams were available (Fig. 4*A*, synergy), thus possibly representing a neural mechanism of multisensory integration. In line with the ongoing debate on the multisensory nature of primary sensory cortices (Ghazanfar and Schroeder, 2006; Liang et al., 2013), cross-modal visuo-haptic interactions leading to enhanced neural representations have been found in the primary somatosensory cortex (S1) (Zhou and Fuster, 2000; Dionne et al., 2010). Here we further characterized these interactions as carrying super-additive/synergistic representations of the active multisensory experience and demonstrated that they are related to the accuracy of active multisensory judgments.

It is also worth noting that our results do not rule out the possibility that other brain areas, not directly related to active sensing, may contribute to regulating the speed and accuracy of active multisensory decisions. Indeed, recent research breakthroughs have explained the development of multisensory representations from different sensory streams in the human brain (Aller and Noppeney, 2019; Cao et al., 2019; Rohe et al., 2019). Furthermore, recent studies have started to investigate how the interactions between sensory representations shape decision formation (Bizley et al., 2016; Franzen et al., 2020; Mercier and Cappe, 2020).

Our primary aim here was to provide the missing link between the active acquisition of multisensory evidence and its transformation to choice. Overall, our findings validated the hypotheses that (1) active sensing guides decision formation via evidence sampling and accumulation and (2) multisensory information spurs perceptual decisions by enhancing the neural encoding of active behaviors. Our information-theoretic analysis also revealed the neural substrates of multisensory interactions in the human brain that support active multisensory perception. Ultimately, we identified and characterized a set of human brain signals that underpin multisensory judgements by subserving an enhancement of the neural encoding of active perception when multisensory information is available.
